# Supplementary material for: Analysis of a Library of Escherichia coli Transporter Knockout Strains to Identify Transport Pathways of Antibiotics
Source: Antibiotics (Basel). 2022 Aug 19;11(8):1129. doi: 10.3390/antibiotics11081129 (PMC9405208; doi:10.3390/antibiotics11081129)
Supplement: Supplementary file 1 [file antibiotics-11-01129-s001.zip › Supplementary Figures.pdf]

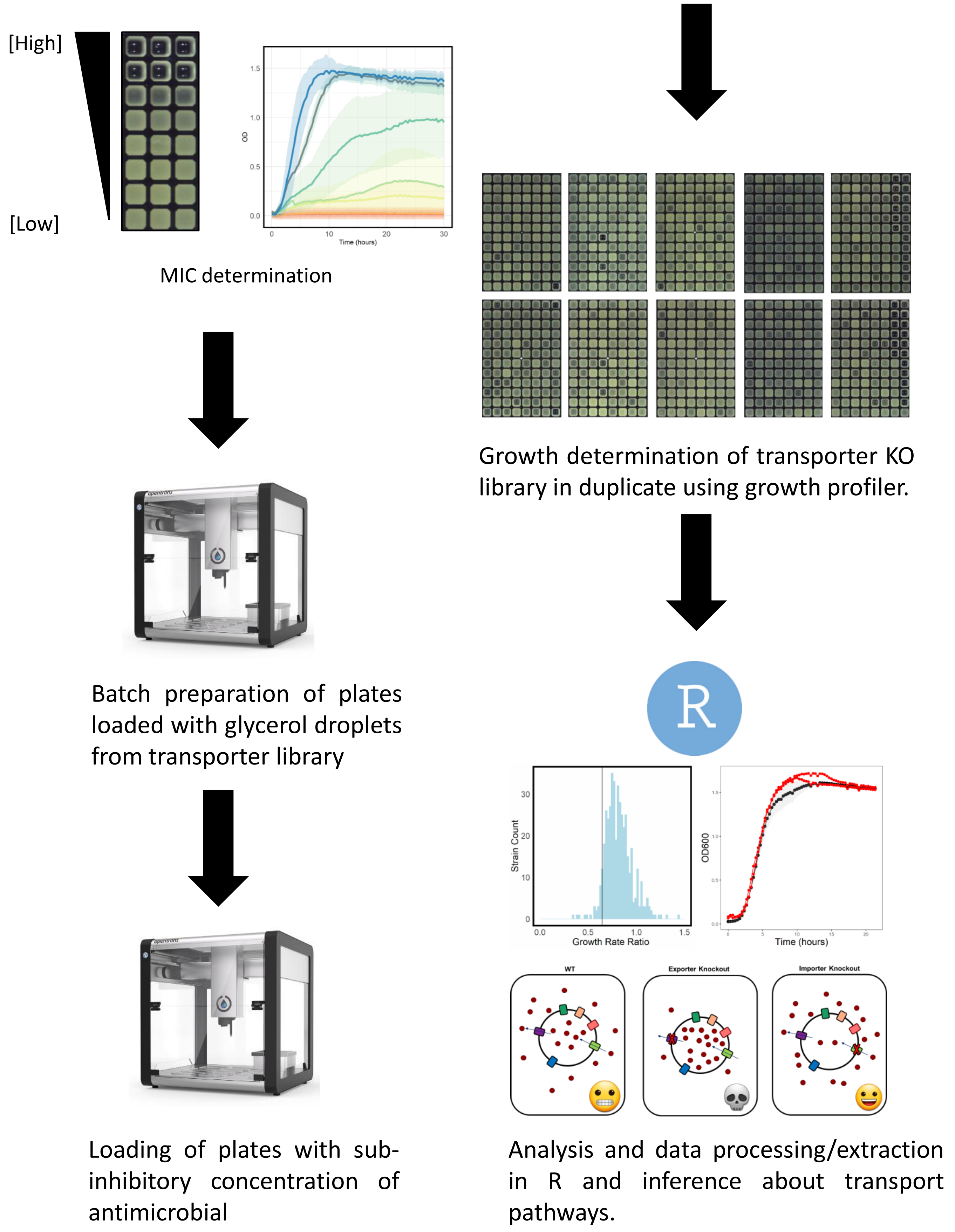

Supplementary Figure S1: Workflow for high throughput screening of transporter knockout library against antibiotics.

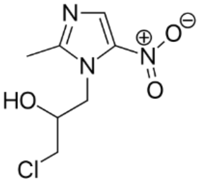

Ornidazole

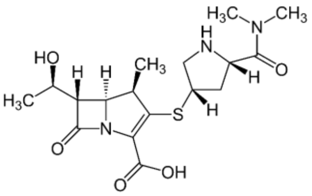

Meropenem

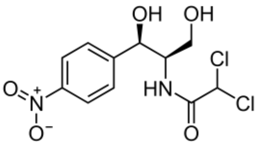

Chlroamphenicol

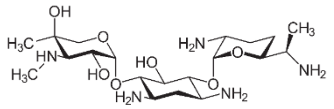

Gentamycin

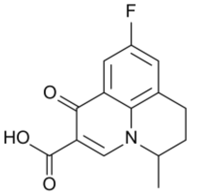

Flumequine

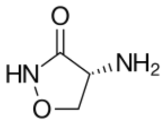

D-Cycloserine

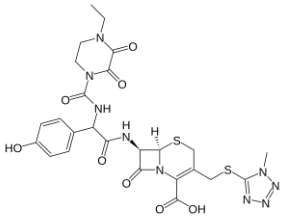

Cefoperazone

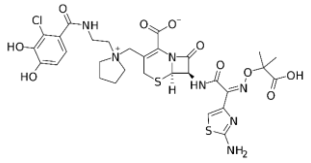

Cefiderocol

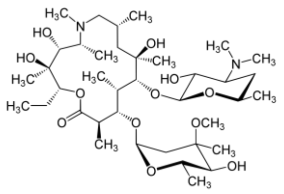

Azithromycin

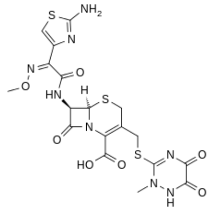

Ceftriaxone

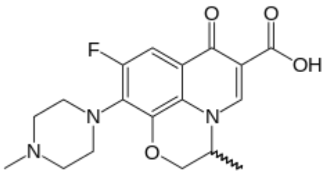

Ofloxacin

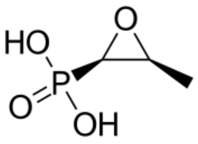

Fosphomicin

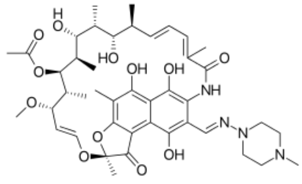

Rifampicin

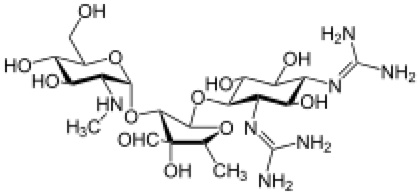

Streptomycin

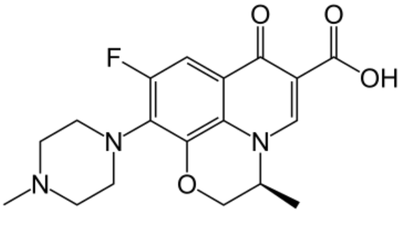

Levofloxacin

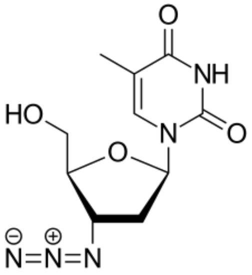

Zidovudine

Supplementary Figure S2: Structures of antibiotics used in this study.
